# Young Children’s Conceptualisations of Kindness: A Thematic Analysis

**DOI:** 10.3389/fpsyg.2022.909613

**Published:** 2022-06-17

**Authors:** Nicole Perkins, Patrick Smith, Paul Chadwick

**Affiliations:** Institute of Psychiatry, Psychology & Neuroscience, Department of Psychology, King’s College London, London, United Kingdom

**Keywords:** children, kindness, altruism and prosocial behaviour, puppet interview, qualitative

## Abstract

Although there is much interest in the development of prosocial behaviour in young children, and many interventions that attempt to cultivate kindness in children, there is a paucity of research exploring children’s lived experiences of kindness and including their voices. In this study, children’s understanding of kindness is approached through qualitative interviews using puppets. Interviews were conducted with 33 children aged 5-6 years in 3 schools in the United Kingdom. Through thematic analysis, 4 themes were developed: (a) doing things for others, (b) relating with others, (c) rules and values, and (d) kindness affects us. These themes are examined in light of current thinking on prosocial and sociomoral development, and several key insights are highlighted, including types of prosocial behaviour, social connection, kindness-by-omission and defending, in-group bias, universal kindness versus personal safety, self-image, and a desire to improve the condition of society. These findings have implications for future research on prosocial development and for the design of kindness-based interventions, as well as providing an ecologically valid method of inquiry for use with young children.

## Introduction

There is growing interest in the study of kindness across the lifespan in both naturalistic and experimental settings, and in the development of programmes that seek to develop kindness. Kindness is an example of prosocial behaviour – that is, voluntary behaviour intended to benefit another (Eisenberg, 1986).

Humans have a remarkable capacity for prosocial behaviour, including kindness. Infants and children begin to show prosocial behaviours such as: sharing food and toys from 8 months (Laible and Karahuta, 2015); informing adults about missing objects by pointing from 12 months (Liszkowski et al., 2006); helping with goal-oriented tasks such as picking up out-of-reach objects, stacking books, opening doors and boxes, and attempting to assist with housework from between 12 and 18 months (Rheingold, 1982; Warneken and Tomasello, 2006, 2007; Svetlova et al., 2010; Dunfield et al., 2011; Dahl, 2015); and providing comfort in response to distress from around 18 months (Svetlova et al., 2010; Dunfield and Kuhlmeier, 2013). They do this spontaneously, anonymously, without rewards, and at a cost to themselves (for example, by disengaging from fun activities), leading some to suggest that humans have a natural predisposition for altruism – prosocial behaviour motivated by a genuine concern for others (Warneken and Tomasello, 2006, 2007, 2009a, 2013a; Warneken, 2013).

Recent research on the foundations of morality in infants raises the possibility that infants may possess a small set of principles that guide their sociomoral reasoning, termed the “first draft” of human moral cognition, which are then shaped by their environment (Graham et al., 2013; Buyukozer Dawkins et al., 2020). These four principles are: fairness, harm avoidance, in-group support, and authority. In a series of experiments using preferential looking, reaching, and violation-of-expectation paradigms, infants seem to expect individuals to treat others fairly (e.g., Buyukozer Dawkins et al., 2019; Geraci et al., 2022), not cause harm to others (e.g., Ting et al., 2019, as cited in Buyukozer Dawkins et al., 2020), and treat in-group members preferentially (e.g., Jin and Baillargeon, 2017), and to expect authority figures to rectify transgressions (Stavans and Baillargeon, 2019). It is proposed that these principles have evolved to support cooperation within small hunter-gatherer communities (Hamlin, 2013; Buyukozer Dawkins et al., 2020).

Although infants and children may be naturally prosocial, when the sociomoral principles that guide their expectations and actions compete with each other, the principles are ranked, with the result that in certain circumstances prosocial behaviour is selective – that is, children are selective about to whom and in what circumstances they expect and exhibit prosocial behaviour. For example, 1.5- and 2.5-year-olds expect fair distribution of resources, except when resources are limited, in which case they expect in-group support to override fairness, with in-group members being treated preferentially (Bian et al., 2018). This makes sense from an evolutionary perspective. We have evolved capacities for prosocial behaviour in order to benefit from an increased likelihood of reciprocal action – if we are kind to others we are more likely to receive kindness back and to benefit in the long run. However, indiscriminate prosocial behaviour would be too costly – if we are kind to everyone, we might not receive enough kindness back to offset the cost – and so we have also evolved selectivity, directing our prosocial behaviour to those most likely to reciprocate. In the absence of direct information about likely reciprocity, such as prior prosocial behaviour, group status can serve as a proxy for potential reciprocity (Hilton et al., 2021).

Research with infants and young children has been extremely helpful in terms of building a picture of the natural prosocial and moral capacities of children. Developmental research on prosociality has tended to focus on the factors that enhance or suppress prosocial tendencies in children, and has been largely experimental and observational (see Brownell, 2013, Laible and Karahuta, 2015, and Martin and Olson, 2015 for summaries). This has allowed researchers to directly observe children’s prosocial responding, rather than relying on ratings of prosociality from children, their parents, or their teachers. This is particularly helpful given that self-reported prosociality may not correlate with actual prosocial behaviour (Böckler et al., 2016). Experimental designs have also allowed researchers to manipulate various conditions to determine relevant antecedents of prosociality. However, the downside of reliance on these methods is that children’s motivations for, and understanding of, prosocial responding are often unknown and can only be inferred from behaviour (Persson, 2005; Eisenberg and Spinrad, 2015). Although motivations for prosocial behaviour may be altruistic in infants and young children – for example, 2-year-olds want to see others helped irrespective of who provides the help (Hepach et al., 2012) – as children age, motivations can range from self-oriented (such as to gain rewards or enhance reputation) to other-oriented, where there is a genuine concern for others’ welfare (Eisenberg et al., 2016). Research with verbal young children that directly seeks their views would allow researchers to better understand young children’s motivations for their prosocial behaviour. There is also a need for research on prosocial development to be more ecologically valid, and for theory to be grounded in children’s everyday lived experience (Binfet and Gaertner, 2015). This research is critical, since the success of interventions designed to cultivate altruism and kindness in children may be dependent on their being both developmentally appropriate and relevant to the real issues that children face in their lives (Greenberg and Harris, 2012; Malti et al., 2016).

Prosociality has been shown to increase wellbeing in adults and children (Aknin et al., 2012; Curry et al., 2018), making it a candidate for public health initiatives aiming to improve societal wellbeing (Galante et al., 2014). Schools are increasingly becoming interested in ways to cultivate social-emotional competencies such as kindness, both to promote individual wellbeing and as a strategy to reduce bullying (Binfet and Gaertner, 2015). Despite this focus on developing kindness in students, there is little research that explores how children conceptualise kindness and that includes children’s voices (Eisenberg and Mussen, 1989; Brownell, 2013; Binfet and Gaertner, 2015). One study surveyed 9-14-year-olds from Canadian schools by asking them to fill in a questionnaire that asked their views on kindness, for example by providing definitions and examples of kindness (Binfet and Passmore, 2019). The authors found predominant themes of helping others (e.g., helping someone who is physically hurt), showing respect (e.g., taking turns), and encouraging others (e.g., giving compliments). Another two studies investigated young children’s conceptualisations of kindness by asking 5-9-year-olds from Canadian schools to draw pictures of kindness (Binfet and Gaertner, 2015; Binfet, 2016). The authors found predominant themes of helping physically and emotionally, giving, including others, acts that maintained friendships, and showing respect. Although these studies provide valuable data from a large number of children, the methodologies of questionnaires and “drawing-telling” (drawing and describing the contents of the drawing) limit the ideas and understandings that children can express, due to the format of the questions, the time restrictions (drawings were limited to 5 minutes), and by limiting young children to examples of kindness that they can draw. A fourth study conducted focus groups with 11-15-year-olds from schools in the United Kingdom (Cotney and Banerjee, 2019). The authors identified 27 themes and grouped these into categories of kind acts (e.g., helping, complimenting, including), situational triggers (e.g., emotional need), psychological goals (e.g., other-focused), impacts (e.g., social), and moderating factors (e.g., social context).

There are currently no studies allowing an open-ended space for young children to express their conceptualisations of kindness, and no studies investigating young children’s conceptualisations of kindness in the United Kingdom.

## The Current Study

In contrast to previous studies on young children’s conceptualisations on kindness (Binfet and Gaertner, 2015; Binfet, 2016), which sampled a large number of children in a briefer format, we wanted to understand the subtleties of how young children understand kindness by sampling a smaller number of children in greater depth. Kindness has been defined in many ways (see Binfet, 2015 for a summary). Some define kindness very simply as “actions intended to benefit others” (Curry et al., 2018). For others, the word “kindness” assumes an altruistic motivation, that is, a genuine concern for others (Cotney and Banerjee, 2019). Kindness has been described as a “baseline altruism”, in other words altruism in all conditions, as opposed to compassion, which is specific to situations of suffering (Wallmark et al., 2013; Gilbert et al., 2019). We chose the term kindness to cover all conditions (i.e., not just situations of suffering), to focus our exploration on other-oriented concern, and to be intelligible to children.

Rather than exploring what factors make children more or less altruistic and examining their behaviour, we were interested in how children make sense of kindness, including what it means to them and what they feel motivates them to act kindly. We do not assume that any ideas that children express about kindness would necessarily lead to prosocial action – that is, we do not suggest that children necessarily do, or can, give an accurate account of how they would behave, or have accurate access to their motivations. Rather, we were concerned with children’s conceptualisations of kindness as a topic of interest in its own right. As little is known about young children’s concepts of kindness, we did not aim to test hypotheses or theory, but rather sought to shed light on what kindness means to children, and what this might tell us about the meanings attached to prosocial behaviour in young children.

We chose to include children aged 5-6 years in the study. By age 5-6, children have a wide repertoire of prosocial behaviours and are increasingly selective about who they help, for example, by directing their helping according to their relationship with the recipient, favouring family, friends, and same-sex peers (Fehr et al., 2008; Olson and Spelke, 2008; Hay, 2009; Moore, 2009; Eisenberg and Spinrad, 2015; Laible and Karahuta, 2015; Martin and Olson, 2015; Lavelle et al., 2017). They are also entering a school environment, where their social worlds are expanding, bringing them into contact with many others. However, they may still be new enough in the school system not to be entirely socialised to its rules and conventions. These factors make for an interesting time developmentally as children grapple with their instincts and competing norms and rules. As such, this age group was selected as a window that may offer rich insights into the topic of kindness from a child’s perspective. Although it may be difficult at any age to access and articulate one’s motivations for acting prosocially, by age 5-6 children may be better able to express their views and speculate on their motivations than children from a younger age group, enabling them to take part in discussions about kindness.

## Materials and Methods

### Epistemological Stance

This study was concerned with what children understand kindness to be, explored through their descriptions of, and conversations about, kindness. We were not aiming to uncover the mechanisms that cause them to *act* kindly (e.g., affective empathy or perspective taking), or that lead them to develop a particular *understanding* of kindness (e.g., parenting practices, socialisation, culture). We did not aim to test existing theory, nor was the study derived from an assumption that prosocial behaviour is innate. Instead, we aimed to conduct an exploratory analysis that places children’s voices and perspectives centre stage. In line with this aim, we conducted an inductive analysis, undertaking a surface reading of the data and taking children’s descriptions at face value. Although children’s realities are undoubtedly constructed, multiple, and subjective, the scope of this study was not to attempt to unravel these realities by attributing meaning beyond the words of the children themselves. We assumed that their talk reflected their reality rather than speculating about what their talk might reveal about how they construct their reality. We adopted a semantic approach to theme generation, in which themes were generated from the explicit meaning of the data, rather than from proposed interpretations of the underlying meaning. We used a bottom-up, data-driven approach to theme generation, using inductive rather than theoretical coding, and taking the raw data as the starting point for coding, rather than existing theory. The analysis focused on data relating to children’s discussions of kindness, rather than aiming to provide a description of all the data. The themes generated represent a broad account of these parts of the data: we were interested in patterns across these parts of the data, rather than the individual stories of participants, in order to address the research question of what children understand kindness to be.

### Grounding the Analysis in the Data

Researchers are always active participants in the meaning-making in analysis, bringing preconceptions and shaping the analysis through their positions and perspectives. Some interpretation is inevitable, and of course necessary, to understand the data. Given that we were conducting an inductive analysis, we aimed to minimise (as far as is realistically possible) imposing our own theoretically-driven interpretations, concepts, or ideas, and to stay as true to the meanings intended by participants as possible. Although it is impossible to free oneself of prior theoretical knowledge and assumptions, we sought to minimise their impact by taking the following practical measures: we used participants’ own words to name codes; the first author engaged with relevant literature only in the later stages of analysis (including literature cited in the introduction of this paper), an approach designed to enhance inductive analyses (Braun and Clarke, 2006); the other authors gave input only at the stage of refining themes, to minimise the influence of their theoretical knowledge; the first author kept a detailed codebook in an attempt to ensure consistency of coding across the data set; and finally the first author generated and answered reflective questions on her expectations about the interviews and themes prior to conducting the interviews, in an attempt to raise her awareness of possible biases that could occur in moderating the interviews and in analysing the data, and in order to record these expectations prior to the analysis. A post-analysis review of the reflective questions indicated that there was little overlap between the first author’s expectations and the final themes generated.

### Design

We adopted a qualitative methodology using interviews, to enable children to bring their unique perspectives to this topic. Through qualitative interviews, participants may be able to bring their experience to bear on the subject matter in an ecologically valid way, adding richness to our understanding of the topic. Interviews also provide an opportunity to observe and participate in the process of meaning-making. Qualitative methods are useful when exploring subjective phenomena such as kindness, as they allow for participant-defined meanings rather than imposing researcher meanings (Willig, 2013). They also allow for participants to be selected based on the richness of the information they can provide (Patton, 2015) and can be a useful complement to quantitative research, offering new perspectives and opening up new areas for further research (Yardley, 2000). Given the exploratory nature of our research question, we chose to use thematic analysis due to its flexible epistemological framework, which allows for integration of concepts of importance to the research question and concepts that are generated from the data through the analytical process (Braun and Clarke, 2006).

We conducted interviews with pairs of children, with the rationale that interviewing in pairs may facilitate conversation in a comfortable way for participants and provide a naturalistic context within which children could share, construct, and negotiate their views together (Lewis, 1992; Hill et al., 1996; Shaw et al., 2011). Although the interviews followed a semi-structured interview schedule, they were conducted flexibly and used play, with interaction based around a puppet (Leon the Lion) who wanted to “learn how to be kind”. Throughout the sessions, Leon (i.e., the interviewer via Leon) asked questions to elicit children’s views and to promote dialogue with and between children on the topic of kindness. We hoped that this interview method would give sufficient space and a context within which children could raise ideas and opinions relevant and important to them. Crucially, we chose to interview children using a puppet (as opposed to the adult researcher directly) based on the suggestion by Shaw et al. (2011). There is a risk that when asked questions by adults, children will assume that adults already know the answer, viewing them as experts, and will therefore seek to give what they feel is the “right” answer, rather than offering their own views. However, where puppets are used, even though children are aware that the adult is operating the puppet, they can enter a realm of play and offer their “expert” advice or opinions to the puppet, who can more easily be positioned by children as having less knowledge than them.

### Interview Process

**FIGURE 1:**
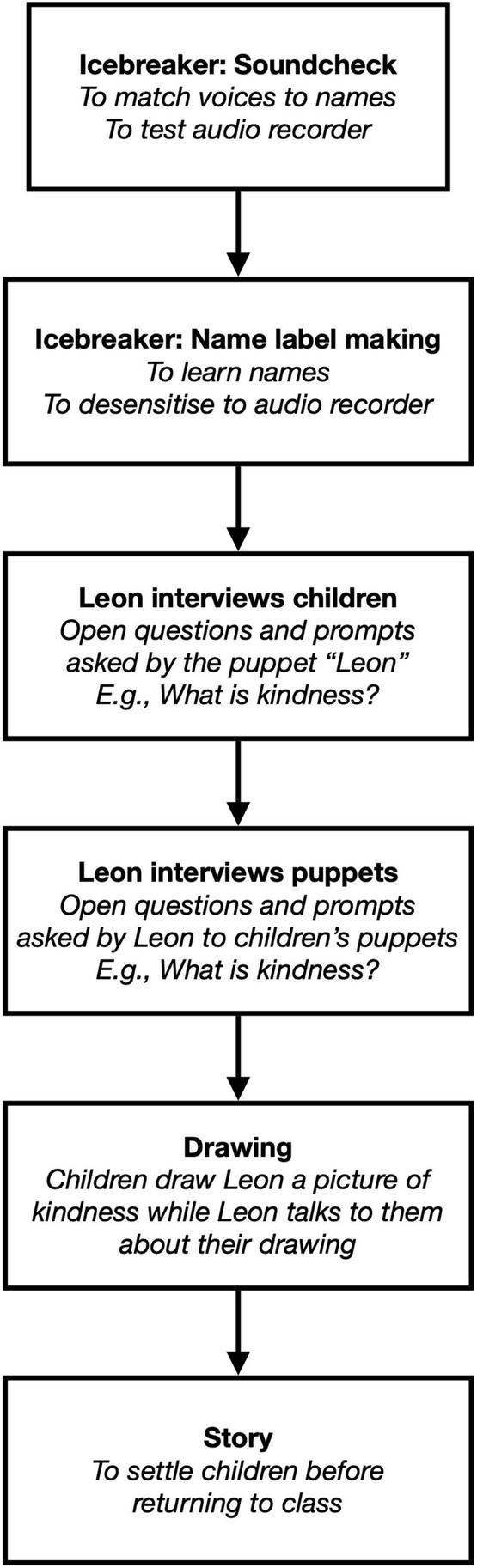
Interview process.

The interviews were one-off, were conducted by the first author, and took place in a classroom that participants were familiar with in their school, with both the interviewer and participants seated on cushions on the floor. The interviewer was not known to the participants prior to the research, and was introduced to the children by first name, along with her “friend”, Leon the Lion. See Figure 1 for a diagram of the interview process. The interviews began with icebreaker activities of a sound check (which allowed voices to be matched to names for transcription and the audio recorder to be tested), and a name label-making exercise (which allowed the interviewer to learn participants’ names, and allowed participants to become more comfortable with the audio recorder running). After the icebreaker activities, Leon (i.e., the interviewer via the puppet Leon) asked open questions and prompts based around four questions: *What is kindness? Should we be kind? What happens if you’re kind? Who should we be kind to?* These interview questions were intended as different ways in to understanding the central question of how children conceptualise kindness, and were selected with the intention of generating discussion, rather than being based on any prior theory. Once no new ideas were apparent, participants were offered a range of puppets that they selected and operated, and Leon asked the same questions of these puppets. The intention here was to facilitate participants to share ideas that, for whatever reason, they may not voice directly, but may be able to voice by speaking through the puppets. Again, once no new ideas were apparent, participants were asked to make Leon a drawing of kindness whilst Leon engaged them in discussion about it (intended as another way to facilitate discussion about kindness), and finally the session closed with a story (intended to settle the participants before their return to class). We included this range of activities both to maintain participants’ interest and engagement, and to provide different possible routes into discussions and perspectives on kindness (Darbyshire et al., 2005). The schedule allowed for both flexibility of topics in order to be led by participants’ discussions, and flexibility of time, so that participants were only taking part in each activity for as long as it seemed they were engaged. Minor adjustments were made to the interview schedule following three pilot interviews. These adjustments were reordering the activities to prioritise those that elicited the most responses (the open questions and prompts), and removing some that did not facilitate discussion (a second story and acting out the drawings participants made). These three pilot interviews were included in the analysis. Sixteen interviews were conducted and audio-recorded with pairs (and one triad) of participating children. Interviews lasted 39 minutes on average and ranged from 27 min to 55 min.

### Participants

A total of 33 participants from three schools in the United Kingdom took part in the research. Participants were aged 5-6 years old. We selected approximately even numbers of boys and girls; 18 were boys and 15 were girls. Class teachers paired children whom they anticipated would feel at ease together and would generate discussion naturally, without one child dominating, and in such a way as to have a mix of male, female, and mixed-sex pairs. Five pairs and one triad were male, five pairs were female, and five pairs were mixed-sex. Twenty-three participants were from Reading, six were from Cambridge and four were from Brighton. We chose these three schools due to their differing levels of mindfulness teaching, which often includes explicit aspects of teaching on kindness and compassion. The three schools represent two contexts: a large, urban, ethnically diverse state school (in Reading), which had no existing mindfulness curriculum and is therefore likely to provide data typical of this age group; and two independent schools (in Cambridge and Brighton) with a predominantly white student population, both of which teach mindfulness to students with the explicit aim of increasing awareness and kindness. Although there is likely to be variety and richness within each of these three schools, we chose to include a smaller number of children from Cambridge and Brighton with the aim to enrich the topic further by capturing potentially more diverse views. Our aim was not to compare children’s responses across the different schools, and therefore we did not analyse the data in this way, but rather to provide a rich analysis of the topic. We conducted purposive sampling, selecting participants whom we believed would provide a range of experiences and insights that spoke to our research question. Our sample was therefore not intended to be representative of a general population, but rather was intended to enable us to conduct a rich analysis of the subject matter. We determined sample size by continuing to recruit participants until saturation of data had been achieved, in other words, until the point at which no new ideas or patterns were being observed in the interviews, and collecting new data no longer sparked new insights. Data were collected in May and June 2015.

Parents were informed of the study by letter and given the opportunity to opt their child out of being invited to take part. No parents opted out of the research and no concerns were raised about the study. Children were then invited to take part by the first author via two puppets: Leon the Lion who wanted to “learn how to be kind”, and Doctor Hattie the Horse who explained the research process. The first author provided age-appropriate information sheets and consent forms and was available to answer questions children had about the research. Children were given one week to decide whether to take part, and participating children provided written consent. Twenty-three out of 30 children in Reading, six out of six children in Cambridge, and six out of six children in Brighton consented to the research. Only four children in Brighton took part, as one child was off school on the day of the interviews and so only two pairs took part.

### Ethical Considerations

The study was approved by the Institute of Psychiatry, Nursing & Midwifery Research Ethics Subcommittee of the King’s College London College Research Ethics Committees (approval number: PNM/13/14-168). Children were invited to take part in the study on an opt-in basis, and this, together with their right to withdraw, was set out in an age-appropriate information sheet and consent form, and was emphasized in the initial talk and at the beginning of each interview. The first author checked that children understood and still wished to participate. Due to the small sample size needed for this study, not all children from the Brighton and Cambridge schools were invited to participate. This could potentially have caused distress if some children had wanted to take part in the research but had not been selected by school staff, and so school staff were encouraged to remind those children not selected and those choosing not to take part that fun activities were going on in their normal classroom. The interviews adhered to good practice guidance when interviewing young children (Shaw et al., 2011).

### Analytic Process

The first author transcribed, anonymised, and analysed the data using thematic analysis. A six-phase process was followed, as outlined by Braun and Clarke (2006). Specifically, data were transcribed for the entire duration of the recordings, in order to capture all relevant data, and transcripts were checked back against the audio recordings to ensure accuracy. The data set was read, initial notes made on codes, and a codebook produced, in order to get a sense of the data set and possible codes before formal coding began. Codes were then collapsed or expanded, and the data set was coded. Data extracts were collated by code, and prevalence of codes recorded at the level of speaker (as opposed to occurrence or interview) to ensure that any conclusions drawn accurately represented the data. Themes and subthemes were then constructed from the codes, and provisionally named. Data extracts were collated by theme, and the constructed themes checked back against both the collated extracts and the entire data set. This iterative process involved continual movement between data, codes, and themes, with themes and subthemes being revised in light of their fit with the data set. The themes were then refined by all authors. This involved the re-categorisation, merging, and renaming of some themes and subthemes. Finally, the entire data set was read again to check that the constructed themes accurately represented the data, and theme names and definitions were finalised. Themes were then examined in relation to relevant literature on prosocial development and to the research question. The analysis was summarised in a report for participants, their teachers, and their parents or guardians.

## Results

In the results section we describe children’s responses and present an analysis of these responses, integrating them with existing theories and experimental work on prosocial behaviour in children. Although we used the word “kindness” when introducing the topic to children, which for some theorists assumes an altruistic motivation, children discussed many different motivations for acting kindly, including self-oriented ones, indicating that, at least in some cases, children were discussing the broader concept of prosocial behaviour.

We constructed four themes from the data: (a) doing things for others, (b) relating with others, (c) rules and values, and (d) kindness affects us. See Table 1 for a description of themes and subthemes. All names of speakers and people referred to in the extracts below have been changed (except the first author’s name). *Interviewer* refers to the interviewer speaking with her own voice, and *Leon* refers to the interviewer speaking through the puppet Leon. Occasionally, we could not identify which child of the dyad or triad being interviewed was speaking, and in these instances we have written *Unknown*. How to report prevalence of themes and subthemes in qualitative analyses is a contentious issue, since although prevalence can give an impression of the strength of a theme or subtheme, it is only one indicator, alongside, for example, emphasis. Rather than reporting quantitative measures of prevalence by speaker, we have used language such as, “most,” “many,” “some,” “a few,” and so on, to give some indication of the prevalence of themes and subthemes across the data set.

**TABLE 1:** Description of themes and subthemes.

Theme	Subtheme	Description
Doing things for others	Giving	Giving things to others (e.g., toys, treats, and presents)
	Helping	Helping others with goal-directed activity (e.g., homework or a game)
	In need	Helping others who are physically or emotionally in need
Relating with others	Being friendly	Friendly dispositional qualities (e.g., smiling and being friendly, loving, and calm)
	Doing things together	Doing things with others (e.g., being with others, playing together, and including others)
	Not hurting others	Not hurting others physically or emotionally (e.g., not hitting, pushing, yelling, being a bully, or being mean)
	Kindness in difficult situations	Responding to bullies, children you don’t like, and people who are being mean, rude, or unkind (e.g., including the bully, defending others, resolving arguments)
Rules and values	Being good is kind	Being “good” (e.g., doing what your teacher or parents tell you to, saying please and thank you, and not “messing about”)
	Being fair	Playing fairly and sharing
	Kindness as a guide	Kindness as a principle to help guide behaviour or decisions
	Kindness is good	Kindness as good in and of itself
	Everyone always	The principles that you should be kind to everyone, you should always be kind, and you should keep trying to be kind despite difficulties
	Challenges and limits to kindness	Challenges to the principle of being kind to everyone (e.g., situations involving bullies, people you don’t like, strangers, and “bad” people)
Kindness affects us	Rewards	Receiving rewards for being kind and punishment for being unkind
	Feelings and self-image	Kindness affects the feelings of the giver and receiver, and the giver’s feelings about themselves
	Making friends	Kindness in order to make friends
	Kindness spreads	The ways in which kindness spreads (e.g., reciprocity and contagion)

### Doing Things for Others

Our first theme included children’s descriptions of kindness that involved *doing things for others*. We grouped their examples into three subthemes: *giving, helping*, and *in need*.

#### Giving

Most of the children in our sample included in their examples of kindness giving things to others, such as toys, treats and presents.

Freddie: And all the, um, Bella’s kind.

Leon: Bella? Is Bella in your class?

Mason & Freddie: *[In unison]* Yeah.

Leon: What does she do that’s kind?

Freddie: Um, every time I have a play date with her she gives me yoyo bears.

#### Helping

Most children gave an example of kindness as helping others with goal-directed activity, such as homework or a game.

Leon: And who do you know who’s kind, Jack?

Jack: My brother.

Leon: Ah, what does your brother do that’s kind?

Jack: He helps me with maths and when I’m reading.

#### In Need

Most children also gave an example of a particular type of helping, namely comforting or looking after others when they are physically or emotionally in need. In the extract below, Thomas and Sophie are deciding on a title for Thomas’s drawing.

Interviewer: Well what would you, what would you say is happening in your picture?

Thomas: Hmm…

Sophie: You could say help people when they get hurt. Or you could at least say help people.

Thomas: Help everybody…

Sophie: In need.

Thomas: …in need.

Children gave a range of examples of ways you could help if someone was in physical or emotional need. These included comforting and looking after them, including them in a game, defending them, helping them up, assessing the damage, nursing the injury, telling a teacher, and accompanying the injured person to seek adult help. These responses all involved doing something to help, but varied in terms of *how* to help. These variations fit with theories of prosocial behaviour that suggest that how children help in situations of need is determined by a variety of factors including personality (e.g., shy children will tend to seek parental or adult help rather than assisting directly; Eisenberg and Spinrad, 2015), and their actual and perceived ability to help in the situation (Eisenberg and Mussen, 1989; Brownell, 2013). Sienna’s suggested response below included checking what was wanted by the person in need, indicating an understanding that different people will have different needs and wants, a role-taking ability that has been observed as early as 15 months in relation to comforting (Hoffman, 1975).

Leon: Do you know anything about kindness?

Sienna: Yes. I know that if someone’s hurt you could give them a cuddle if they want one.

Leon: Ah, you could give someone a cuddle if they’re hurt.

Sienna: Only if they want one. If they don’t, don’t give them one.

The three subthemes of *giving, helping*, and *in need* appear to map onto three types of prosocial behaviour commonly delineated in the literature: *sharing, helping*, and *comforting* (Paulus and Moore, 2012; Dunfield, 2014). Research suggests that these three types of prosocial action involve different antecedents, mechanisms, and motivations (Paulus, 2014, 2018), have different developmental trajectories (Svetlova et al., 2010; Brownell, 2013), and are underpinned by different neural mechanisms (Paulus et al., 2013; Chakroff and Young, 2015). The children in this study often gave examples related to only one or two of these three subthemes, consistent with the finding that different types of prosocial behaviour do not necessarily correlate, in other words, that children may understand or display certain types of prosocial behaviours and not others (Dunfield et al., 2011; Dunfield and Kuhlmeier, 2013; Dunfield, 2014; Eisenberg and Spinrad, 2015). These examples of kindness are similar to those found in previous research with young children, where “helping physically,” “helping emotionally,” and “giving” were predominant themes (Binfet and Gaertner, 2015; Binfet, 2016). In addition to sharing, helping, and comforting, other types of prosocial behaviour have been proposed, such as informing (conveying useful information) and cooperating (working towards a shared goal; Warneken and Tomasello, 2007, 2009b). However, the children in our sample did not describe informing or cooperating as examples of kindness.

### Relating With Others

Our second theme captured children’s examples of kindness that involved how we *relate with others*. We constructed four subthemes: *being friendly, doing things together, not hurting others*, and *kindness in difficult situations*.

#### Being Friendly

Most of the children in our sample mentioned dispositional qualities when discussing kindness, including observable behaviours and internal states, such as having a kind voice, smiling, and being friendly, loving, and calm.

Unknown: And I know some girls who is kind.

Leon: Ah, which girls?

Unknown: Oh I know some.

Unknown: Imogen.

Leon: Ah, and what does she do that’s kind?

Unknown: Well she…

Unknown: She like, she’s quiet and calm and like hey, caring, like she be’s nice to me and be’s polite…

#### Doing Things Together

Our second subtheme includes discussions of kindness as a social way of being, for example, being with others, playing together, and including others. In contrast to the first theme, what was important here was not doing something for others, but simply being with others. This social element was talked about as kind in itself by most of the children in our sample.

Leon: So what… what do you do to be kind to your friends?

Gracie: You can, you can, you can, you can play games with one another, and sing…

Phoebe: Try not to leave people out?

Gracie: …and sing songs.

Phoebe: And if it’s a one or two player game and there’s three of you can, you can make up a, um, an or a just a completely different character.

Many of the examples of kindness in our subthemes *being friendly* and *doing things together* have been found in studies with older children that have collated children’s examples of prosocial behaviour through prototype questioning, questionnaires, and focus groups – namely: complimenting, including others, being friendly, affectionate, humorous, happy and cheerful, calm, willing to play, and not rude (Bergin, 2015; Binfet and Passmore, 2019; Cotney and Banerjee, 2019). In studies with younger children, themes of including others, playing with others, and being affectionate have been reported (Binfet and Gaertner, 2015; Binfet, 2016). Although there is some discussion of social interaction in theoretical literature on prosocial development, this is mainly in the context of the pleasure of social interaction being a *motivator* for prosocial behaviour (Paulus, 2014; Eisenberg et al., 2016), rather than social interaction as a *type* of prosocial behaviour. For the children in our sample, simply being with someone can be a kind act.

#### Not Hurting Others

Most children also described kindness as not hurting others, physically or emotionally, for example, not hitting, pushing, yelling, being a bully, or being mean.

Leon: Kitty, do you know anything about kindness? I’m trying to learn.

Jessica: *[Speaking through a puppet]* Yes.

Leon: What do you know?

Jessica: I know, I know how to be *really* kind.

Leon: Do you? Oh, please tell me. I’d like to be really kind.

Jessica: You don’t hurt someone. That’s the first thing you don’t do.

Leon: Ah, okay, so I mustn’t hurt anyone. Okay, I’ll remember that. What else?

Jessica: You don’t bully anyone.

Leon: Okay, so don’t hurt anyone, don’t bully anyone.

Jessica: Don’t push anyone over.

Leon: Okay, I won’t push anyone over.

Jessica: And that’s all I know.

This emphasis on non-harming fits with infants’ sociomoral expectations that, all else being equal, individuals should not harm each other (Buyukozer Dawkins et al., 2020), and with evidence that 3-year-olds avoid helping others who have previously harmed another (Vaish et al., 2010), and that 4-year-olds distribute fewer resources to characters who have previously harmed others when resources are scarce (Kenward and Dahl, 2011). Research on prosocial behaviour in children tends to focus on commission; for example, helping, sharing, comforting, and informing (Warneken and Tomasello, 2009b; Dunfield, 2014). However, the children in our sample gave many examples of kindness-by-omission: behaviours that it is kind *not* to engage in. Bergin (2015) provides a list of 24 prosocial behaviours observed in school-aged children, which includes five examples of kindness-by-omission: not fighting, not hurting others’ feelings, not making fun of others, not bragging, and not yelling.

#### Kindness in Difficult Situations

Our final subtheme included discussions about how to respond kindly in difficult situations: those involving bullies; children you don’t like; and people who are being mean, rude, or unkind. Most children discussed this issue, with suggestions for how to respond ranging from including the bully in the game, sticking up for those being bullied, trying to resolve the argument, telling a teacher, walking away, and hurting them back.

Polly: Also, um, also, also Leon, you, you also have to be kind because um, the good thing is that if someone ever says something a little bit rude to you then just say okay, and just walk away.

Although infants show a preference for prosocial over antisocial characters (Hamlin et al., 2007; Hamlin and Wynn, 2011), this preference is reversed when the antisocial behaviour is directed at those who have previously harmed another (Hamlin et al., 2011), suggesting that moral retribution is deeply rooted and may serve to maintain cooperative systems within communities (Hamlin, 2013). Bergin’s (2015) list of prosocial behaviours also includes standing up for others, confronting wrongdoers, and acting as a peacemaker, and defensive and intervening action has been proposed as a further subtype of prosocial behaviour (Geraci and Franchin, 2021). Consistent with the differing types of defending identified by Geraci and Franchin (2021) – for example, solution-focused, reporting to authority, and aggression – the children in our sample gave different examples of ways that one could respond to antisocial behaviour. These differing responses could reflect age-related and developmental differences. For example, as children get older they tend to reduce physical aggression and increase their protesting and tattling (reporting misbehaviour to an authority figure) as a way of defending others (Ingram, 2014).

The subthemes presented here, particularly the *not hurting others* and *kindness in difficult situations* subthemes, could provide avenues for future research with children of this age, whose social worlds are beginning to expand at school, since the issues identified by the children in this study are arguably crucial for harmonious classrooms and communities. Kindness-by-omission and defensive behaviour as an act of kindness are two specific areas that may benefit from further research.

### Rules and Values

Our third theme covered children’s discussions of the rules and values that relate to kindness. We grouped these ideas into six subthemes: *being good is kind, being fair, kindness as a guide, kindness is good, everyone always*, and *challenges and limits to kindness*.

#### Being Good Is Kind

Most children gave an example of kindness that could be termed “being good” or “compliance”, such as doing what your teacher or parents tell you to, saying please and thank you, and not “messing about”.

Leon: So how can I be kind? What can I do to be kind?

Unknown: Um, be good when you’re going to the doctor?

Leon: Ah, how can I be good going to the doctor?

Unknown: Um…

Gracie: You have to stay put in the car, you can’t wriggle around.

Phoebe: No. No, it’s not the car. The room that… the…

Gracie: Oh, don’t touch anything.

Phoebe: Don’t touch anything or don’t move on the feet? Cos um… when I go to the doctor I have to sit in a seat that goes up and maybe down and stuff like that….Oh no that’s, that’s the dentist I’m now talking about….Don’t wriggle otherwise it will be a different, difficult for the doctor do, to do stuff.

Gracie: Or the dentist.

This subtheme touches on an important distinction between compliant and spontaneous prosocial behaviour, which have been found to have differing antecedents and consequences and do not necessarily correlate (Eisenberg and Mussen, 1989; Eisenberg and Spinrad, 2015; Imuta et al., 2016). Similarly, Persson (2005) differentiates requested and self-initiated prosocial behaviour and found no significant relation between them.

#### Being Fair

In their discussions about kindness, around half of our sample raised the idea of fairness, for example, sharing, having the same amount of things like toys or sweets, and playing fairly.

Phoebe: Well so I suppose she is kind but well, she wasn’t in those days when we played my little pony not rainbow rocks.

Leon: Ah so nowadays, what does Lily do that’s kind?

Gracie: Plays it fairly and lets us be in charge of it, all of us.

Research with infants suggests that sociomoral principles of fairness are present from as early as 4 months, with studies showing that 4-, 10-, and 19-month-olds expect resources to be distributed equally (Sloane et al., 2012; Meristo et al., 2016; Buyukozer Dawkins et al., 2019), that 1.5- and 2.5-year-olds expect fair distribution of resources when resources are plentiful (Bian et al., 2018), that 9-month-olds prefer characters who intend to distribute fairly rather than unfairly (Geraci et al., 2022), and that 21-month-olds detect a violation when two individuals are rewarded equally when only one of them had contributed to completing a chore (Sloane et al., 2012). Research with children suggests that fairness is an important guiding principle of their prosocial behaviour. For example, children aged 2-5 years know they ought to be fair, even if their cognitive abilities are not yet developed enough to implement this (Chernyak et al., 2016), 3-year-olds share with peers after collaborating on a task (Warneken et al., 2011), 4-year-olds prefer to distribute resources evenly rather than according to the recipient’s past behaviour or their relationship to the recipient (Kenward and Dahl, 2011; Flook et al., 2019), 5-6-year-olds distribute resources unequally in order to rectify inequality (Rizzo and Killen, 2016), 6-year-olds punish unfair behaviour and sacrifice personal gain to ensure fair distribution between others (McAuliffe et al., 2015), 6-8-year-olds throw away a resource that they could have kept for themselves to avoid inequity (Shaw and Olson, 2012), and 7-8-year-olds favour equality over increasing joint gain (Fehr et al., 2008). For the children in our sample, principles of kindness seem to be tied to sociomoral principles of fairness.

#### Kindness as a Guide

In a few instances, kindness was discussed as a principle which, if followed, could help guide behaviour or decisions, suggesting the use of kindness as an internalised principle or moral value (Eisenberg and Mussen, 1989; Eisenberg et al., 2016).

Leon: So what, what happens when you’re kind to someone?

Gracie: Um they start being friends with you?

Phoebe: If you, if you didn’t be kind la… um before, they’ll be, you have to be kind…

Gracie: …again…

Phoebe: …and then you know what not to do.

#### Kindness Is Good

A few children described kindness in a way that suggested they understood it as “good” in and of itself.

Interviewer: So is there anything else that you wanted to tell Leon that you didn’t get a chance to yet?

Aisha: Well, I think helping your friend it’s very good and kind.

#### Everyone Always

When discussing who you should be kind to, most children’s initial response was that you should be kind to family and friends.

Leon: Hello Chipmunk. I’m Leon the lion. Do you know anything about kindness?

Amelia: *[Speaking through a puppet]* Yeah.

Leon: What do you know?

Amelia: You can be kind to your friends and your parents.

A sociomoral expectation of in-group support has been found in infants. For example, 17-month-old infants expect others to help in-group, but not out-group, members (Jin and Baillargeon, 2017); 18-month-olds expect non-harming of in-group members but not out-group members when there is no provocation, and when there is provocation, they expect in-group members to be harmed much less than out-group members (Ting et al., 2019, as cited in Buyukozer Dawkins et al., 2020); 13-month-olds expect harm to in-group but not out-group members to be punished by withholding help (Ting et al., 2019); and 1.5- and 2.5-year-olds expect in-group members to be treated preferentially when resources are limited (Bian et al., 2018).

This initial, often immediate, response from children in our sample that kindness should be directed towards kin and in-group supports the notion of an in-group bias in prosociality in this age group (Hay, 2009; Eisenberg and Spinrad, 2015; Laible and Karahuta, 2015; Martin and Olson, 2015), and fits with experimental evidence that children aged 2 to 9 show more prosocial behaviour towards friends, family, and in-groups (in some cases even towards minimal groups created in the lab) than towards strangers, non-friends, and out-groups (Fehr et al., 2008; Olson and Spelke, 2008; Moore, 2009; Dunham et al., 2011; Paulus and Moore, 2014; Benozio and Diesendruck, 2015; Flook et al., 2019; Hilton et al., 2021), that children’s moral reasoning is contingent on the group status of those involved (Decety and Cowell, 2014), and that children aged 5-13 believe there is a greater obligation to help racial in-groups (Weller and Lagattuta, 2013).

However, most children then expanded this principle to others, and to everyone. This alternative response may reflect a learnt norm or principle (Eisenberg et al., 2016). The shift from children’s initial response that kindness ought to be directed towards one’s in-group, to the view that it ought to be universal, may reflect the shaping of innate sociomoral principles according to environment and culture (Graham et al., 2013).

Leon: And who should we be kind to?

Vedant: Everyone.

Leon: Everyone?

Zainab: Even Mrs Jones the Headteacher.

Leon: Even Mrs Jones the Headteacher. *[Laughs].*

Vedant: Even people in Japan and Spain.…and Portugal.

A related principle emphasised by some children is that you should *always* be kind. When giving examples of kindness, these children placed importance on always doing these things. For example, always say sorry when you hurt someone and always say please and thank you. As one child put it:

Jacob: …kindness you should always do even if you um, when you’re about eighty-two.

You should also keep trying to be kind despite difficulties, for example when others are unkind:

Leon: And what if somebody’s not very nice to you, should you be kind to them?

Zainab: Yeah.

Vedant: You should…

Zainab: Still. It doesn’t mean you should, you can stop!

Vedant: Like if…

Leon: Ah.

Zainab: It still means you can do it!

Leon: Okay.

Vedant: And don’t! You have to…

Zainab: And they be mean to you…

Vedant: …not give up. And try again and again and again and again and if it doesn’t work you have to still try again and again and again and still try again and again and again.

Leon: Keep on trying to be kind?

Zainab: Of course.

#### Challenges and Limits to Kindness

Although there was a strong sense that you should always be kind to everyone, certain scenarios posed a challenge to this principle, with some children debating whether the principle was appropriate in these situations and some opinions existing in tension with the *everyone always* subtheme. These were scenarios involving different recipients of kindness: bullies, people you don’t like, strangers, and “bad” people.

Leon: So I’ve got one more question, who should I be kind to?

Unknown: Yourself.

Unknown: *[Laughs]*

Leon: Kind to myself?

Unknown: Everyone.

Leon: And everyone.

Unknown: Not bullies though.

There are many studies showing that prosocial behaviour in children is affected by the characteristics of the recipient (see Martin and Olson, 2015 for a summary), and this theme highlights the importance of considering who the recipient is when exploring aspects of prosocial behaviour (Padilla-Walker and Carlo, 2015). As with the *everyone always* subtheme, these examples reflect an in-group bias and provide rich descriptions of the challenges young children perceive in acting prosocially towards out-group members.

Leon: Yes, well my last question is, who should I be kind to?

Benji & Isaac: *[In unison]* Everyone.

Leon: Everyone?

Benji: Hmm.

Isaac: Except for robbers.

Leon: Except for robbers. Ah, so I should be kind to everyone except for robbers.

Isaac: Yep.

Benji: Cos they might steal your stuff.

Phoebe: You don’t help strangers cos if they’re pretending to be a stranger and they’re actually a burglar they might um…

Phoebe & Gracie: *[In unison]* …take you away…

Unknown: …from your family.

A few children argued that you only need to be kind to “good” people and that you can be unkind to “bad” people.

Leon: What about somebody I don’t know?

Unknown: Ur…

Unknown: Mm, no.

Leon: No? Why not?

Aaliyah: They might be a baddie.

Leon: Ah, they might be a baddie.

Esme: And you won’t know if they’re bad.

Leon: Ah, okay. And what about mm, what about one of the children I don’t know in this school? In a different year? Should I be kind to them?

Unknown: Mm…

Aaliyah: Um, only if they’re good.

This fits with infants’ early-emerging tendencies to prefer characters who act negatively towards antisocial individuals (Hamlin et al., 2011), and with experimental evidence that young children, when forced by uneven resources, give more to prosocial individuals (Kenward and Dahl, 2011), that 4-10-year-olds believe “villains” and bullies are less worthy of moral concern (Neldner et al., 2018), and that elementary and high school children are more likely to believe that people should help family and friends rather than disliked or stigmatised others (e.g., criminals; Eisenberg and Spinrad, 2015). However, a few other children reasoned that you should be kind even to bullies, people you don’t like, and strangers.

Leon: And should I be kind to people I don’t know?

Jacob: Yes.

Logan: Um, I think so.

Jacob: Yes! You still should.

Leon: Logan, what do you think?

Logan: No, I always run away and scream.

Leon: Ah, okay.

Jacob: I think you should do it um, anyway….Because they will be nice to you if you do it to them.

The divergence of views among children supports the idea that prosocial behaviour to in- and out-groups constitute different types of prosocial behaviour (Padilla-Walker and Carlo, 2015). Children’s capacity for moral reasoning has been linked to their beliefs about obligations to help disliked others (Eisenberg, 1983), with greater capacity for moral reasoning allowing children to reflect on their prosocial and moral values and to overcome competing tendencies such as in-group bias (Davidov et al., 2016). As children develop, their reasoning becomes more complex, simultaneously taking into account sociomoral principles, such as equality and equity, as well as group membership and loyalty (Rutland and Killen, 2017). The discussions between children in these interviews highlight some of the dilemmas within such moral reasoning. Understanding individual differences in the range of recipients to whom children direct their kindness is an important area for future research (Spinrad and Eisenberg, 2017).

The discussions in the *everyone always* and *challenges and limits to kindness* subthemes illustrate the conflicts between innate sociomoral principles and learnt norms, as well as the weighing-up of different rules – for example, be kind to everyone versus don’t talk to strangers (providing examples of both the value-based and self-protective motivations described by Eisenberg and Spinrad (2015)) – and the weighing up of the costs and benefits to the self associated with acting kindly – for example, personal gain through reciprocity versus personal safety. The children’s discussions about safety also support the idea that children make assessments about friendliness, helpfulness, and trustworthiness when making decisions about whether to act prosocially (Brownell, 2013), and that fear-proneness could be a barrier to acting prosocially towards strangers (Laible and Karahuta, 2015).

### Kindness Affects Us

Our final theme captures ideas about how kindness affects the receiver, the giver, and society in general. We grouped these ideas into four subthemes: *rewards, feelings and self-image, making friends*, and *kindness spreads*.

#### Rewards

Some children explicitly raised the possibility of receiving rewards for being kind and punishment for being unkind, consistent with theories of motivations for prosocial behaviour, which include avoiding punishment, obtaining rewards and approval (Eisenberg et al., 2016), and strategically weighing up costs and benefits (Eisenberg and Mussen, 1989; Böckler et al., 2016).

Leon: Ah okay. Do you think I should be kind, Sophie?

Sophie: Yeah.

Leon: Why?

Sophie: Cos like it will save some other people’s energy and you might get rewards for it.

However, experimental work with children ranging from 20 months to 11 years shows the opposite: that rewards do not correlate with prosocial behaviour, and can even undermine it (Fabes et al., 1989; Grusec, 1991; Warneken and Tomasello, 2008). The children in our sample who raised this possibility may believe rewards to be a reason for being kind, but whether rewards do in fact motivate prosocial behaviour in 5-6-year-old children would need further testing empirically.

#### Feelings and Self-Image

Most children reported that kindness affects the feelings of both the person receiving kindness and the person giving kindness.

Leon: Hello Plum and Big Mouth. I’m trying to be a kind lion. Can you help me?

Polly: *[Speaking through puppets]* Yes.

Leon: What can I do to be kind?

Polly: Well, if someone maybe is all alone you could smile to them and they might feel a little more cheery.

One child’s statement implied that he believed the feelings of others affect your own feelings.

Unknown: You’ll be happy that you’ll be, that, that you’ve be happied someone.

Finally, some children made comments that implied that whether or not you are kind affects your feelings about yourself, positively or negatively:

Leon: Ah, I see. So, how will I feel if I’m kind to people?

Sienna: Um, you will sort of feel um, happy yourself if you be kind to people you’ll be proud of yourself for being kind.

The effects of kindness described here by children (feeling happy or proud when being kind, and feeling bad when being unkind) are consistent with findings that 1-5-year-olds show happiness when acting prosocially (Aknin et al., 2012, 2015), and that 3-6-year-olds may be motivated to act prosocially by the anticipation of happiness (Paulus and Moore, 2017). The children’s responses also fit with theories of prosocial motivation that include increased feelings of self-esteem due to acting in accordance with internalised values (Eisenberg et al., 2016), and guilt due to not acting prosocially (Bandura, 1977; O’Connor et al., 2012; Eisenberg and Spinrad, 2015; Davidov et al., 2016; Malti et al., 2016; Vaish et al., 2016).

#### Making Friends

About half of our sample discussed making friends as an effect of kindness.

Leon: Should I be kind?

Esma: Yeah.

Aaliyah: Yeah.

Leon: Should I? Why?

Esma: So you can get as many friends as you can.

The reason for wanting many friends was made explicit by one child.

Unknown: …if your other friend, if your friend didn’t want to play with you there is another friend, that, that, that means if you got another um, friend you can play with them.

This motivation to be kind in order to make more friends was also apparent in some children’s discussions of out-group members. In the extract below, Thomas and Sophie are discussing whether to be kind to children you don’t like.

Leon: Should I be kind to them?

Thomas: Yeah.

Leon: Should I? Why?

Thomas: Um, because, because if *they* get hurt you should just help them, then, and then they can make friends with you…

Sophie: Then, and they might…

Thomas: …and then it’s like you can make friends and then basically it’s how you make more friends.

The desire for social interaction has been proposed as one potential motivator of prosocial behaviour (Eisenberg and Mussen, 1989; Paulus, 2014; Eisenberg et al., 2016). Social interaction is thought to be inherently pleasurable, especially for young children, and therefore some prosocial behaviours may be brought about not by a genuine concern for others, but by a motivation to interact socially with others (Paulus, 2014). Eisenberg et al. (2016) highlight that, although social interaction is often an *outcome* of prosocial behaviour, there is little direct evidence for it as a *motivator* of prosocial behaviour. The prevalence, in these interviews, of children’s reasoning about kindness involving the explicit aim of making friends and having friends to play with provides some initial evidence for the desire for social interaction as a motivator of prosocial behaviour. Eisenberg et al. (2016) suggest that in addition to the pleasure of relating to others, children may behave prosocially in order to enhance existing friendships, and that this could partially explain why children are motivated to help those closest to them and those they like, rather than those they dislike. However, the discussions with some of the children in our sample suggest a different picture: that children may strategically choose to be kind to those they dislike, in order to transform the relationship into one of friendship, with the ultimate goal to have more friends to play with.

#### Kindness Spreads

This final subtheme relates to children’s discussions about how kindness spreads through its effects. First, about half of our sample raised the idea that if you are kind, you get kindness back.

Leon: Ah, okay. So what, what would happen if I’m kind to somebody?

Sienna: They might be kind back to you.

Jessica: And they might help you.

Sienna: When you’re hurt or something.

One child even implied that reciprocity was obligatory.

Leon: Hello Breathe Fire. My name’s Leon. Do you know anything about kindness?

Abdullah: *[Speaking through a puppet]* Yes! If, if you must, you must be, be kind to anyone who wants to be kind to you.

This “law of reciprocity” was often implicit as an underlying assumption in children’s discussions of kindness.

Leon: So I should be kind to them?

Phoebe: Mmm only if you want them to be kind which we don’t really go near them anymore so I wouldn’t go near them?

This suggests reciprocity is a well-accepted motivation among children of this age, in line with evolutionary explanations of prosociality (Hilton et al., 2021) and with research showing that children act on a principle of reciprocity as early as 3 years old (Olson and Spelke, 2008; Warneken and Tomasello, 2013b), and that 5-year-olds give more when recipients are aware of their giving (Leimgruber et al., 2012) and when observed by someone who is able to reciprocate (Engelmann et al., 2013). The data in the current study suggest both strategic (if I’m kind, they’ll be kind to me) and norm-based (you must be kind back) reasoning about reciprocity. Second, some children suggested that you can pass kindness on by being kind. In the extract below, Mason and Freddie are discussing children that they don’t like.

Leon: Should I be kind to them?

Mason: Yes.

Freddie: Yeah?

Leon: Why?

Mason: Because if you…

Freddie: They might, it, they, you’ll teach them, if you being kind you’ll teach them how to be kind, by being kind!.All, all you have to do to teach them to be kind is be kind.

Mason: So, if you’re, you be kind you can show someone else that how to be kind, and you’re, and so like if there’s someone you don’t really like in your class, ur, class cos they like, don’t really like you, you could, you could be kind to them and then, and then you will teach them how to be kind.

Freddie: If we teach you, tea… you teach them.

Unknown: And them can teach another person…

Unknown: Like a circle, like…

Unknown: I, we teach you, then you teach them!

Unknown: And then them teach another them!

Unknown: And then it keeps on going on.

Unknown: Until, like infinity.

In several interviews, children demonstrated that kindness can be taught by being kind, by highlighting to Leon when they had done something kind in the interview.

Logan: That’s being kind. Just to let you know, Leon.

Leon: Oh, what, what is, Logan?

Logan: Tidying up the pens.

These first two ways of spreading kindness – reciprocity and passing it on – fit with theoretical models and empirical findings that people tend to engage in direct reciprocity (helping those who have helped them) and generalised reciprocity (“paying forward” help that they have received from one person to a different person; Melamed et al., 2020). Our interviews show that some children are already reasoning about these two forms of reciprocity as early as 5-6 years old. Reputational giving (giving more in the presence of those who may reciprocate) and reputational rewarding (rewarding those who have given to others; Melamed et al., 2020) were not mentioned by the children in our sample, suggesting that, although 5-year-olds may act to enhance their reputation (Engelmann et al., 2013), they may not yet engage in these more sophisticated forms of prosocial reasoning. Third, some children indicated that kindness can change people’s behaviour.

Leon: Okay, and what do you think, Ethan? Should you be kind to bullies?

Ethan: Not really….Well, sometimes, quite a lot you should.

Edward: Yes. Yes you should because…

Ethan: Yeah, because then they’d lend a hand.

Edward: Um, cos then they, they could change…

Ethan: Make friends.

Edward: …they could change their behaviour. Change their life!

And finally, two children implied that you can expand your existing kindness to new people, extending your instinctual in-group prosociality towards everyone, including members of the out-group.

Leon: Who should I be kind to?

Polly: Well, everybody really, because everybody has to be kind to other people, so you have to be kind to Nicole and also you have to be kind to other people too, like me and Lucy, and you’re already very kind so you can do it to other people too.

These discussions of how kindness spreads to others suggest that concern for the condition of society as a whole might be a motivator of kind behaviour for some of the children in this sample (Eisenberg and Mussen, 1989).

## Discussion

### Summary

We interviewed 5-6-year-old children about their views on kindness, making this the first study to directly seek young children’s perspectives on this topic in an in-depth way. The majority of existing research has focused either on observational and experimental methods with infants and children who are too young to articulate their views, or has sought the views of older children and adolescents. As such, our study is unique in providing insights into young children’s conceptualisations of kindness and their motivations for engaging in kind behaviour. Our analysis led to eight key insights: (1) the types of prosocial behaviour discussed by children mapped onto three types commonly delineated in the literature, namely sharing (*giving*), helping (*helping*), and comforting (*in need*), lending support for these as distinct types of prosocial behaviour; (2) children placed a high value on friendships and social connection, an area that has received relatively little attention in the literature, and that could be viewed both as a *type* and *motivator* of prosocial behaviour, as well as being an outcome of that behaviour; (3) kindness may also include kindness-by-omission – what *not* to do – and defending or intervening, whether by peacemaking, tattling, or aggressive action; (4) a natural in-group bias in prosociality may compete with an ideal of universal kindness; (5) a value-based principle of universal kindness may compete with self-protective concerns about personal safety, particularly in situations that involve kindness towards strangers who may pose a threat; (6) the differing responses children gave regarding prosocial behaviour towards in- and out-groups supports the notion of these as distinct types of prosocial behaviour; (7) children view kindness as affecting both the receiver and the giver, and one’s self-image; and (8) improving society may be a motivator for prosocial behaviour in some children.

Similar to Binfet and Gaertner (2015) and Binfet (2016), the children in our sample gave examples of kindness that included helping physically and emotionally, giving, being friendly, and including others. Our study builds on this previous work by allowing an open-ended space for children to discuss their conceptualisations of kindness. This is reflected in the analysis, which accords with previous findings but moves beyond them by capturing the more nuanced understandings of kindness amongst children of this age; for example, the dilemma between being kind to strangers versus staying safe. Although previous studies (Binfet and Gaertner, 2015; Binfet, 2016) identify the theme “acts that maintain friendship” (e.g., saying, “thank you for playing with me”), it is difficult to infer motivation from these examples. Our methodology gave children the opportunity to explicitly discuss their motivations, which included being kind in order to have more friends. The children in our sample also discussed ideas about kindness that have been found in previous research with older children, but not before in this age group. The 11-15-year-old adolescents in Cotney and Banerjee’s (2019) study discussed: the inappropriateness of approaching strangers, reciprocity and contagion as effects of kindness, being happy and proud as emotional impacts of kindness, and making or maintaining friendships as a goal of kindness. Our study is the first to show that these more complex ideas, which are present in adolescence, are already present in some children as young as 5 or 6. The children in our sample did not mention the topics of forgiveness, honesty, volunteering, or an acceptance of others’ differences in relation to kindness, themes that have been reported in work with older children (Binfet and Passmore, 2019; Cotney and Banerjee, 2019), suggesting that these are more complex ideas beyond the developmental stage of 5-6-year-olds.

### Reflections on the Analysis

Both the interviews and analysis will have been shaped in various ways, for example: by interviewing the children in pairs as opposed to individually; by the use of puppets and the presence of the adult interviewer; by interviewing within the school setting as opposed to at home or in another context; by broader social and cultural norms and values; by the position, interests, and prior knowledge of the researchers; and by the language that the interviewer used to elicit responses. In addition to these influences on the data, there is a further level at which meaning is being constructed, as children develop and form their understandings through the process of social engagement and discussion (Vygotsky, 1980; Eisenberg and Mussen, 1989; Svetlova et al., 2010). The children in this study may have been moving into their zone of proximal development in terms of their understanding of the topic, through the process of the interviews alongside their peers. The interviews in this study should not, therefore, be seen as simply collecting understandings that are already present and known, and the themes and insights described should not be seen as “truths” that have been uncovered through the analysis. Rather, the interviews themselves may form part of the development of understanding, even creating new understanding in those children taking part as they make meaning together. There are possible indications of this process in the data: on many occasions children copied each other, sometimes even making eye contact and speaking slowly in order to speak in unison, and so reaching a shared position or articulation; children queried each other’s perspectives, debating the issue until an agreed position was reached; and the prevalence of codes by speaker indicates a clustering within interviews around particular examples of kindness, suggesting that children influenced each other within the interviews. All these observations suggest that the children were learning together and that their understanding, rather than complete and pre-existing, was at least in part emerging through dialogue. The puppet interviewing method used in this study may therefore be of interest to researchers exploring proximal development in other areas of child development.

The examples of kindness that children gave may further illuminate the process of learning. Some appear “top-level” and could represent the learning of principles and norms from adults or peers, and may reflect the higher-level motivations (such as adherence to internalised principles) mentioned by Eisenberg et al. (2016).

Amelia: It’s, it’s something that you, that you, that you help people and care about people.

In contrast, other definitions appear grounded in everyday action and experience, with the beginnings of abstraction, suggesting that the meaning of a concept like kindness may also be derived from experience.

Isaac: Well I know not to splash at people….And also um, not to kick, um, into them when you’re swimming.

Many children defined kindness through examples of its opposite: unkind behaviour (e.g., the *not hurting others* subtheme), suggesting that one may also learn what kindness is by understanding or encountering what is unkind.

Jessica: Kay. First thing I, I know about kindness is you can’t hurt anyone.

### Strengths

Strengths of the current study included the use of puppets as a method for interviewing children. This led to an abundance of data as children were extremely keen to contribute and to interact with the puppets. The use of puppets also appeared to facilitate children being honest and sharing views they were less certain about.

Zainab: *[Speaking through a puppet]* I’m still… I’m a bit feel nervous, because I don’t actually know all about kindness.

The method also appeared to be enjoyable and engaging for children, an ethical aim of research involving children (Shaw et al., 2011).

Vedant: I want to have another private talk with Nicole again.

Lucy: I wish I lived with Leon.

Other strengths of the study included the attempts to minimise bias, for example the first author answering reflective questions prior to interviewing and engaging with theory only in the later stages of analysis. Given the data-driven approach adopted here, the parallels between the themes constructed and pre-existing theory suggest that our themes may not be unique to this sample nor heavily influenced by the researchers’ biases.

### Limitations

Kindness is an attitude, behaviour, and disposition that is normally encouraged in children, even taught to them at home and school. When children are questioned about kindness by an adult, then, it is possible that children believe there is a “right” answer that the adult is seeking. In this study, we attempted to minimise this experimenter-pleasing bias by questioning children using a puppet (rather than an adult interviewer questioning the children directly), and this seems to have been effective to some degree. However, it is possible that some children still attempted to respond in a way that they believed was wanted or required of them.

### Future Work

In researching prosociality and designing interventions to promote prosocial behaviour, it is important to consider both the type of prosocial behaviour and the target of that behaviour, for example, in-group versus out-group (Padilla-Walker and Carlo, 2015). The present study provides detailed descriptions of how children conceptualised these categories, and may be helpful for designing interventions that map onto children’s existing understanding. Kindness-by-omission and defending are types of prosocial behaviour that warrant further research. The motivations for prosocial behaviour that children expressed in this study may also inform further research and intervention development. For example, understanding the importance of friendships and social connection as both a *type* and *motivator* of prosocial behaviour may help with designing effective interventions that resonate with the actual concerns and desires of children.

In-group bias is a much-researched phenomenon, and has important implications for efforts to cultivate kindness and universal prosocial behaviour. However, the in-group out-group distinction in prosocial behaviour in children, and how to cultivate prosocial behaviour towards out-group members, is under-researched (Eisenberg and Spinrad, 2015). In addition to sociomoral principles of in-group support being part of a “first draft” of moral cognition (Graham et al., 2013), children may continue to develop their selective prosociality as an evolved strategy to protect them from exploitation (Warneken and Tomasello, 2009a). An unfortunate consequence of these evolved strategies is that behaviour towards our out-group can (in certain situations) be exclusionary or harmful (e.g., Fehr et al., 2008; Benozio and Diesendruck, 2015). For example, as well as preferring characters that help rather than harm similar others, infants also prefer characters who harm rather than help dissimilar others (Hamlin et al., 2013). Although selective prosociality may help small communities to function, in modern, large-scale, diverse societies, limiting kindness to those closest to us causes serious problems, as we have the ability to help or harm not only our immediate in-group, but people around the world, and are faced with global threats such as pandemics and climate change, which necessitate working together to find solutions (Böckler et al., 2018). There may therefore be a need for interventions to widen the circle of kindness, expanding our natural capacities for kindness to be more inclusive and to counter our evolved biases (Lavelle et al., 2017). In-group bias in prosociality is thought to increase from age 3 to 8 (Fehr et al., 2008), therefore this age bracket could be a particularly important developmental period in which to introduce such interventions. The findings of the current study suggest important considerations for this work. In most interviews, value was placed on universal kindness, with some children going beyond the dyadic implications of reciprocity and placing value on the spread of kindness through society and, eventually, the world. The children in this study also gave rich descriptions of their concerns about the risks and limits of universal kindness. The success of future interventions may depend on incorporating an understanding of the real-life issues such as these that children perceive.

In order to further develop our understanding of how children conceptualise kindness, gender and age differences could be examined using the methodology of the current study. This would similarly help to inform the design and implementation of interventions aiming to cultivate prosocial behaviour. Our puppet method may only be appropriate up to the age of around 7, but the same principle of asking children to teach an interlocutor, for example, an avatar, could be utilised as a way to generate rich data with older children.

Given the success of this method for interviewing young children, future research could make use of it in any area where the topic of interest involves the views of children. It may be a particularly helpful method for exploring topics where children perceive adult researchers as more knowledgeable than them, or where they feel their views may not be acceptable (e.g., topics that are perceived to have a moral dimension), and which are therefore at risk of experimenter-pleasing bias. From an ethical perspective, it is also a useful method for ensuring that the data collection process is enjoyable for children, rather than placing a burden on them.

### Conclusion

We investigated young children’s views on kindness using a novel method of interviewing and a qualitative methodology. This approach yielded new insights and offered support for several theories in the current literature from the perspectives of children, using an ecologically valid method. The puppet method used in this study was particularly successful in engaging 5-6-year-old children, and provided a wealth of data.

### Implications

The findings from this study highlight potential areas for future research on prosocial development, namely: social connection; kindness-by-omission and defending; the dilemma of universal kindness versus safety; in-group bias; the effects of kindness on feelings and self-image; and a desire to improve society. The puppet method used may also be helpful to researchers working with young children and looking for novel ways to engage children and explore their views. When designing interventions to cultivate prosocial behaviour in children, several important factors warrant consideration, including the type of prosocial behaviour, the target of that behaviour, the context, and the varying motivations that children may have. It is hoped that the insights from this study may inform intervention development by providing a useful perspective from which to consider these issues.

## Data Availability Statement

The raw anonymised data supporting the conclusions of this article will be made available by the authors, without undue reservation.

## Ethics Statement

The studies involving human participants were reviewed and approved by the Institute of Psychiatry, Nursing & Midwifery Research Ethics Subcommittee of the King’s College London College Research Ethics Committees. Written informed consent from the participants’ legal guardian/next of kin was not required to participate in this study in accordance with the national legislation and the institutional requirements.

## Author Contributions

NP, PS, and PC designed the study. NP collected and analysed the data and wrote the manuscript. PS and PC gave input into the final stages of data analysis. All authors commented on previous versions of the manuscript and read and approved the final manuscript.

## Conflict of Interest

The authors declare that the research was conducted in the absence of any commercial or financial relationships that could be construed as a potential conflict of interest.

## Publisher’s Note

All claims expressed in this article are solely those of the authors and do not necessarily represent those of their affiliated organizations, or those of the publisher, the editors and the reviewers. Any product that may be evaluated in this article, or claim that may be made by its manufacturer, is not guaranteed or endorsed by the publisher.
